# From microbial dynamics to risk prediction: a nomogram-based model for hospital-acquired infections in rehabilitation settings

**DOI:** 10.3389/fcimb.2026.1723835

**Published:** 2026-02-17

**Authors:** Bangying Yu, Yunping Guo, Chenchao Wu, Meidan Fang, Weiwei Ma, Ali Li, Minhua Zheng, Jing Wang

**Affiliations:** 1 Taizhou Hospital of Zhejiang Province Affiliated to Wenzhou Medical University, Taizhou, Zhejiang, China; 2 Department of Health Management Center, Enze Hospital, Taizhou, Zhejiang, China

**Keywords:** external validation, hospital-acquired infection, nomogram, rehabilitation department, risk stratification

## Abstract

**Objective:**

To analyze microbial infection patterns and develop a predictive model for hospital−acquired infection (HAI) in rehabilitation inpatients.

**Methods:**

A retrospective cohort study included 635 patients admitted between August 2018 and February 2025; 4,523 clinical specimens were analyzed. After exclusions, 361 patients were classified into HAI (n=213) and non−HAI (n=148) groups. Significant variables from univariate analysis were incorporated into LASSO and logistic regression to build a prediction model, which was visualized as a nomogram. A simplified scoring tool and a web application were developed. External validation was performed using 332 patients from three hospitals.

**Results:**

Among 4,523 specimens from 635 rehabilitation inpatients, the overall positivity rate was 61.2%. Sputum cultures were most frequent, while urine cultures increased over time. Key pathogens like *Klebsiella pneumoniae* and *Pseudomonas aeruginosa* showed distinct temporal trends. High antimicrobial resistance was prevalent, especially among multidrug-resistant organisms, with carbapenem-resistant Enterobacteriaceae being the most common MDRO type. Regression analyses identified age, prothrombin time, D−dimer, and C−reactive protein as key risk factors of HAI, while albumin and high−density lipoprotein cholesterol were protective.The nomogram demonstrated good discriminatory ability and calibration internally (AUC = 0.741) and maintained robust, generalizable performance in external validation, with the simplified risk score achieving an AUC of 0.799, while also showing stable performance before and during the COVID-19 pandemic. The tool is publicly accessible at: https://wjing-enzemed.shinyapps.io/hospital-infection-risk-en/.

**Conclusion:**

Our findings elucidate key microbiological patterns and predictive factors for HAI in rehabilitation inpatients. The developed model, utilizing readily available clinical parameters, shows robust and generalizable performance in stratifying infection risk, which can aid early intervention and optimize resource allocation in rehabilitation care.

## 1 Introduction

Rehabilitation patients represent a distinct clinical population characterized by advanced age, multiple comorbidities, immunosuppression, frequent invasive procedures, and prolonged hospitalization. These factors collectively contribute to a significantly elevated risk of Hospital-acquired infections (HAI) (Hagen et al., 2025). HAI exert profound impacts on patient outcomes, with particularly severe consequences in immunocompromised individuals, often leading to extended hospital stays, increased healthcare expenditures, and substantial socioeconomic burdens (Chéron et al., 2010; Kreitmann et al., 2024).

In rehabilitation settings, HAI predominantly manifest as respiratory infections (Biscevic-Tokic et al., 2013), catheter-associated urinary tract infections (Jitpratoom and Boonyasiri, 2023), surgical site infections (Abou-Hatab and El-Bahnasawy, 2013), and secondary skin/soft tissue infections resulting from pressure ulcers (Olivo et al., 2020). Severe cases may progress to life-threatening bloodstream infections. Current diagnostic approaches exhibit notable limitations, as traditional methods relying on overt symptoms and microbiological evidence frequently result in delayed interventions, particularly for patients with neurological impairments who often present with atypical symptoms (Maezawa et al., 2016). The epidemiological landscape is further complicated by the rising prevalence of multidrug-resistant organisms, including MRSA, CRE, and MDRB, with key risk factors encompassing severe underlying conditions, corticosteroid use, hypoalbuminemia, prolonged hospitalization, and invasive procedures such as tracheostomy, central venous catheterization, and indwelling urinary catheterization (Zhang et al., 2021; Yang et al., 2025).

Biomarkers serve as critical tools for HAI risk assessment, providing objective measures of inflammatory response intensity (e.g., CRP, PCT, IL-6/8/10, sTNFR1, sTREM1) and infection progression (Zhu et al., 2021; Yang et al., 2023). While dynamic monitoring systems like APACHE II and SOFA scores (McCarthy et al., 2022) (Pawar et al., 2021) effectively evaluate organ dysfunction and mortality risk, existing frameworks inadequately account for rehabilitation-specific high-risk factors such as neurological dysfunction, prolonged bed rest, and invasive procedures. Consequently, there is an urgent need to develop personalized prediction models integrating multidimensional data—demographics, comorbidities, functional status, and laboratory parameters—leveraging machine learning algorithms to enable early risk stratification and address the limitations of conventional tools in infection control.

This study will retrospectively analyze clinical and laboratory data from rehabilitation inpatients, employing logistic regression to identify key risk factors for HAI and construct predictive models. The findings aim to provide clinicians with evidence-based support for precision prevention strategies.

## 2 Methods

### 2.1 Study cohorts and Study design

A retrospective cohort of 635 rehabilitation inpatients who underwent microbial culture at Taizhou Hospital (Zhejiang, China) between August 2018 and February 2025 were enrolled. Demographic data, clinical information, imaging results, and laboratory parameters were collected, including complete blood count, blood biochemistry, arterial blood gas analysis, coagulation tests, and infection markers.

Positive rates and bacterial species were compared across sample types (cerebrospinal fluid, bronchoalveolar lavage fluid, sputum, urine, blood) stratified by hospitalization duration, and antimicrobial resistance patterns were analyzed. Patients were excluded if they had pre−existing infection at admission (n=10), latent infection (n=38), colonization without active infection (n=204), or lacked routine laboratory data within 48 h of admission (n=22).

The remaining 361 patients were categorized into a hospital−acquired infection (HAI) group (n=213) and a non−HAI group (n=148). Clinical and laboratory indicators at admission were compared between groups. Statistically significant variables were entered into univariate logistic regression and LASSO regression to identify HAI risk factors. A predictive nomogram was developed and its performance was rigorously evaluated. To facilitate clinical use, a simplified risk−scoring form and an online web application were created.

**Figure 1:**
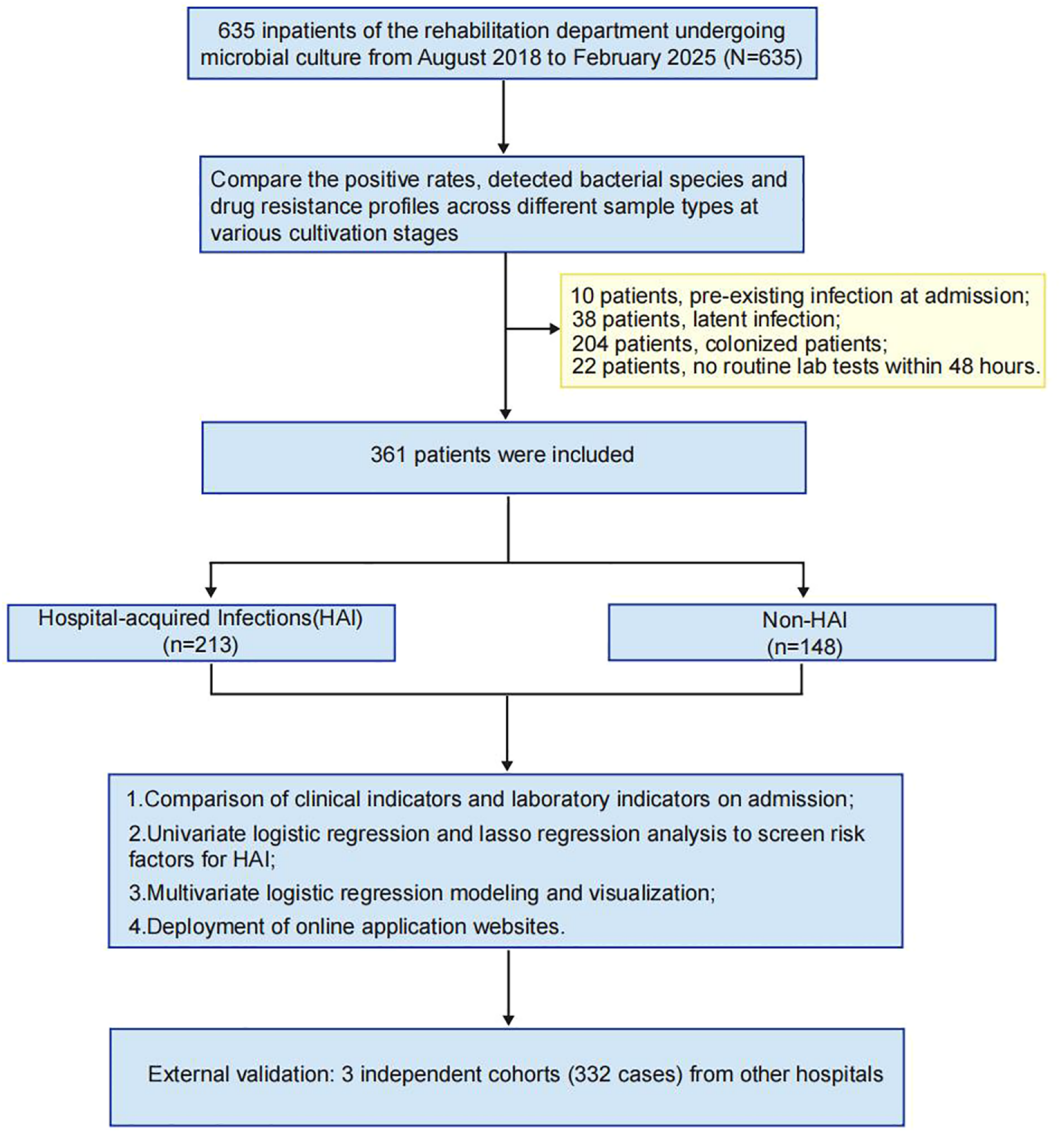
Patient inclusion and exclusion flowchart. A total of 635 inpatients undergoing microbial culture were initially screened. After applying the exclusion criteria, 361 patients were included in the final analysis.

External validation was performed using 332 patients from three independent rehabilitation departments: Enze Hospital (n=282), Wenzhou TCM Hospital of Zhejiang Chinese Medical University (n=26), and Jiaxing Second Hospital (n=24) (**Figure 1**).

### 2.2 Diagnostic criteria

Hospital−acquired infection (HAI) is defined as infection that was neither present nor incubating at admission and occur more than 48 hours after hospitalization, with microbiological confirmation requiring: for specimens from sterile sites (e.g., blood, cerebrospinal fluid), a positive culture supported by new-onset clinical symptoms or radiological evidence of infection; for specimens from non-sterile sites (e.g., sputum, urine), a positive culture must be accompanied by new-onset, clear clinical signs and/or radiological findings attributable to the infection site, and the clinical decision to initiate targeted antimicrobial therapy based on this assessment serves as strong supporting evidence.

Colonization is defined as an isolated positive culture in the absence of any new attributable clinical symptoms, signs, imaging findings, or consequent antimicrobial treatment.

The non-HAI group comprised patients with no evidence of any active infection throughout their entire hospitalization, after rigorously excluding infections present or incubating within 48 hours of admission.

### 2.3 Culture specimen collection and quality assessment

All culture specimens were collected prior to the administration of antibiotics.

#### 2.3.1 Cerebrospinal fluid culture

Cerebrospinal fluid samples were obtained by physician-performed lumbar puncture under strict aseptic conditions. Minimum collection volumes were 1 mL for adults and 0.5 mL for children, with at least 2 mL collected when tuberculosis or cryptococcal infection was suspected. Samples were placed in sterile screw-cap tubes and transported to the laboratory immediately, with a target delivery time of ≤15 minutes at room temperature. If transport was delayed, samples were maintained at 35°C and refrigeration was strictly avoided. Specimens were accepted for culture if they were clear or slightly turbid without visible contamination. Samples were rejected if they were, clotted, refrigerated, or if transport exceeded one hour.

#### 2.3.2 Blood culture

Blood samples were collected following strict skin disinfection using a combination of alcohol and either iodine or chlorhexidine. Venipuncture was performed at the onset of fever or, ideally, before antibiotic administration. For adults, 8–10 mL of blood was inoculated into each culture bottle, while for children, 1–5 mL was used per bottle. Each set of cultures required both aerobic and anaerobic bottles. Blood was not routinely drawn from intravascular catheters unless catheter-related bloodstream infection (CRBSI) was specifically suspected. Specimens were accepted based on adequate filling volume, absence of clotting, and adherence to proper collection technique. Samples were rejected if bottles were underfilled, if only a single bottle was submitted per set, or if the culture bottle was expired or damaged.

#### 2.3.3 Sputum culture

Sputum samples were obtained by deep-cough expectoration after a morning mouth rinse and collected in a sterile cup. Specimens were delivered to the laboratory within two hours of collection. If a delay was anticipated, samples were refrigerated at 4°C for a maximum of 24 hours. Specimen quality was assessed microscopically. Samples were accepted for culture if they contained >25 white blood cells (WBCs) and <10 squamous epithelial cells (SECs) per low-power field (LPF), corresponding to a WBC-to-SEC ratio of >2.5:1. Specimens were rejected as unsatisfactory if they showed salivary contamination (SEC >10/LPF) or contained no WBCs.

#### 2.3.4 Bronchoalveolar lavage fluid culture

BALF samples were collected via bronchoscopic lavage using 20–60 mL of sterile saline pre-warmed to 37°C. A fluid recovery of at least 30% was targeted. The lavage fluid was collected into a sterile container. Transport to the laboratory was required within one hour. If immediate transport was not possible, samples were stored at 4°C for a maximum of 24 hours. Acceptable specimens typically appeared turbid or milky, with macrophage-predominant cytology. Samples were rejected if they were grossly bloody (indicating significant trauma with red blood cells >10%) or if they were stored unrefrigerated for more than two hours.

#### 2.3.5 Urine culture

Urine specimens were collected via clean-catch midstream voiding, catheterization, or suprapubic aspiration (considered the gold standard). Samples were placed in a sterile cup. Transport to the laboratory was required within two hours of collection. If a delay occurred, specimens were refrigerated at 4°C for no longer than 24 hours. Specimens were accepted if they were collected using a sterile method and showed no signs of gross contamination. Samples collected by non-sterile methods (e.g., bag urine from infants), stored at room temperature for over two hours, or visibly contaminated were rejected.

### 2.4 Instruments and reagents

Complete blood count and CRP were measured using the Mindray BC-6800 Plus automated hematology analyzer and its corresponding reagents. Biochemical indicators were analyzed using the Beckman Coulter AU5800 automated biochemistry analyzer and its corresponding reagents. Coagulation function were tested using the STAGO STA-R Max automated coagulation analyzer and its corresponding reagents. PCT was detected using the Roche Infinity electrochemiluminescence analyzer and its corresponding reagents. Blood gas analysis was performed using the Siemens RAPIDPoint 500e blood gas analyzer and its corresponding reagents.

### 2.5 Missing values management

Variables with a missing data proportion of ≤40% were screened and retained by calculating the missing rate for each variable. Multiple imputation was performed on the remaining missing values in the screened dataset using the random forest method (meth = ‘rf’) from the mice package. The specific parameters were set as follows: 5 imputed datasets were generated (m = 5), with a maximum of 50 iterations (maxit = 50), and a random seed was fixed (seed = 500) to ensure reproducibility. The fifth imputed dataset was extracted and saved as the complete dataset for subsequent analysis.

### 2.6 Statistical analysis

Data analysis and visualization were performed using R software. Continuous variables were expressed as median (25th-75th percentile) and compared between groups using the Mann-Whitney U test. Risk factors for HAI were identified through univariate logistic regression and lasso regression analyses. The predictive model was visualized using a nomogram, and its performance was evaluated using calibration curves, clinical impact curves, and ROC curves. A P-value <0.05 was considered statistically significant.

## 3 Results

### 3.1 Temporal distribution and antimicrobial resistance patterns of microbial cultures in rehabilitation inpatients

**Figure 2:**
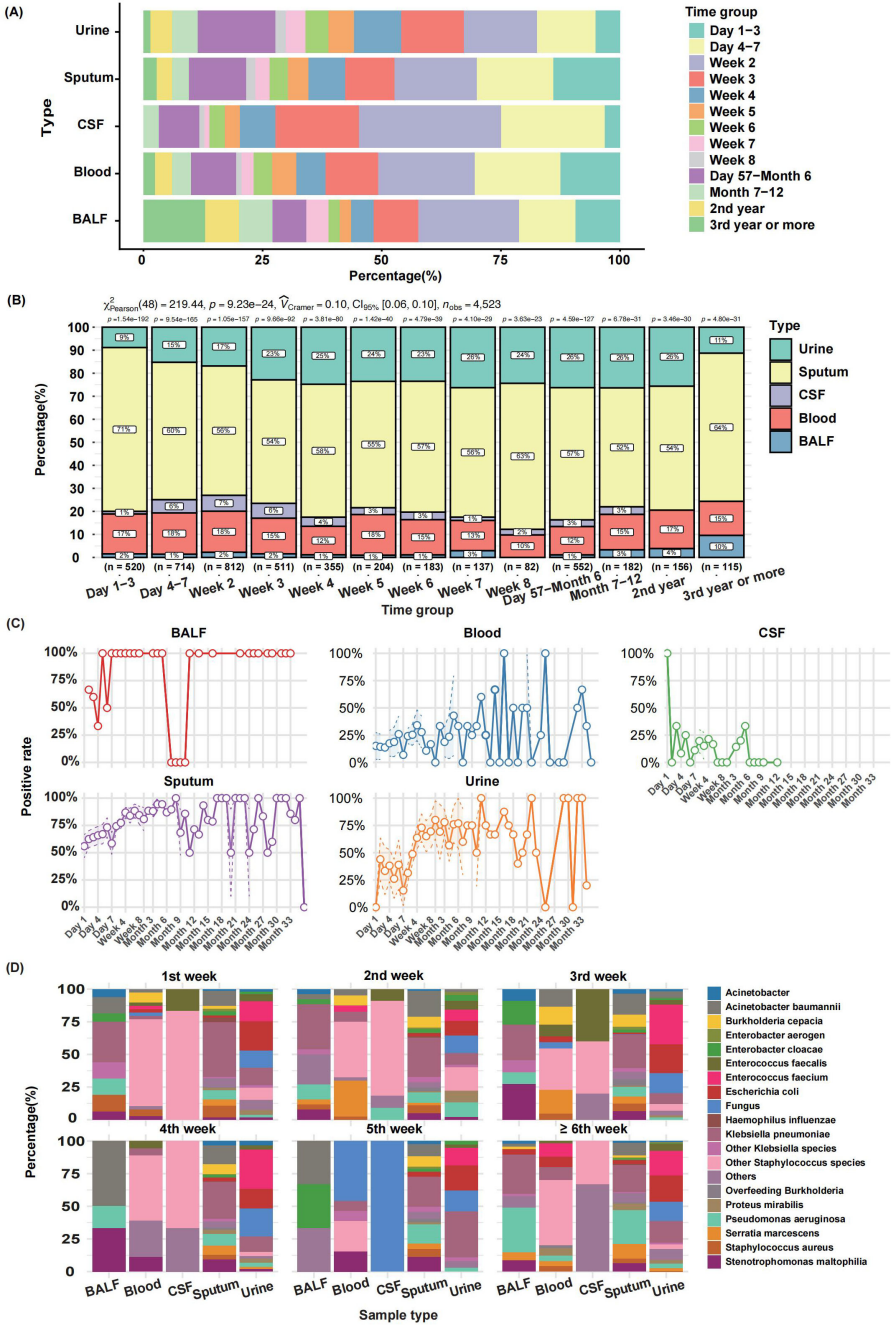
Temporal Distribution and Dynamic Analysis of Microbial Culture Positivity in Rehabilitation Inpatients. **(A)** Sampling time distribution of different culture types. **(B)** Composition ratio of sample types at different stages. **(C)** Dynamic positive rate of different culture sample types. **(D)** Microbial culture dynamic changes of various sample types at different stages.

A total of 4,523 clinical specimens—including cerebrospinal fluid, bronchoalveolar lavage fluid, sputum, urine, and blood cultures—were collected from 635 inpatients, of which 2,770 yielded positive results (positivity rate: 61.2%). Except for urine samples, approximately 50% of specimens were obtained within the first two weeks of admission. Sputum cultures constituted the majority throughout hospitalization, while the proportion of urine cultures increased markedly after the third week (**Figures 2A, B**).

At admission, positivity rates varied by specimen type: 100% for cerebrospinal fluid, ~70% for bronchoalveolar lavage fluid, ~50% for sputum, ~20% for blood, and 0% for urine. During hospitalization, sputum and bronchoalveolar lavage fluid consistently showed the highest positivity rates (sputum averaging ~80% and remaining largely positive except at 6–12 months). Blood culture positivity fluctuated between 20% and 50% in the first six months before rising thereafter. Urine culture positivity increased from ~30% within four weeks to ~75% in later periods (**Figure 2C****).**

Microbiological analysis of positive cultures revealed distinct temporal trends. In bronchoalveolar lavage and sputum samples during the first week, *Klebsiella pneumoniae* predominated, accompanied by *Acinetobacter baumannii, Pseudomonas aeruginosa*, and *Staphylococcus aureus*; the prevalence of *Pseudomonas aeruginosa* increased progressively over the hospitalization period. Blood cultures showed dynamic shifts: *Staphylococcus* spp. dominated in week 1, *Serratia marcescens* emerged in weeks 2–3, and *Klebsiella pneumoniae, Stenotrophomonas maltophilia*, and fungi became prominent in weeks 4–5. Cerebrospinal fluid consistently yielded *Staphylococcus* spp. and *Enterobacter aerogenes*. Urine cultures initially displayed diverse Enterobacteriaceae, transitioning to dominance by *Enterococcus faecium* and *Escherichia coli* with prolonged hospitalization (**Figure 2D****).**

Among isolated pathogens, the most frequently identified bacteria included Enterobacteriaceae (mainly *Escherichia coli* and *Klebsiella pneumoniae*), *Stenotrophomonas maltophilia, Staphylococcus hominis, Staphylococcus epidermidis, Staphylococcus capitis, Staphylococcus aureus, Pseudomonas aeruginosa*, and *Haemophilus influenzae*. Enterobacteriaceae exhibited high resistance rates to ampicillin and ticarcillin/clavulanic acid. *Enterococcus* spp. showed elevated resistance to multiple agents, including ampicillin, penicillin G, ciprofloxacin, clindamycin, levofloxacin, moxifloxacin, nitrofurantoin, and trimethoprim-sulfamethoxazole. *Staphylococcus aureus* demonstrated high resistance to penicillin. Other coagulase-negative staphylococci (e.g., *Staphylococcus epidermidis, Staphylococcus capitis*) were frequently resistant to penicillin, cefoxitin, ciprofloxacin, clindamycin, erythromycin, levofloxacin, and oxacillin. *Pseudomonas aeruginosa* displayed high resistance to ticarcillin/clavulanic acid and imipenem (**Supplementary Figure 1A****).**

Compared with isolates from non-MDRO carriers, those from MDRO carriers exhibited significantly higher resistance rates to Penicillin (87.9% vs. 78.1%), Ampicillin (81.2% vs. 71.8%), and Oxacillin (79.1% vs. 43.6%). The overall resistance rates did not differ significantly across specimen types (e.g., sputum, urine, blood; *P* = 0.280). In this cohort, carbapenem-resistant Enterobacteriaceae (CRE) were the most prevalent MDRO type in all sample categories, with isolation rates ranging from 27.6% to 62.5%, followed by methicillin-resistant *Staphylococcus aureus* (MRSA), which was isolated at rates between 3.9% and 12.5% (**Supplementary Figures 1B–D****).**

### 3.2 Comparison of clinical features and admission laboratory parameters between HAI and non-HAI patients

A comparison of clinical characteristics and laboratory parameters at admission revealed several key distinctions between patients with and without HAI. While no significant differences were found in sex distribution or comorbidities, patients with HAI were significantly older (median age: 65 vs. 59 years; P < 0.01) and had a substantially longer median hospital stay (46 vs. 29 days).

Laboratory profiles also differed markedly. Patients with HAI presented with elevated levels of white blood cell count, neutrophil-to-lymphocyte ratio (NLR), aspartate aminotransferase (AST), blood glucose, serum chloride, creatine kinase (CK), lactate dehydrogenase (LDH), prothrombin time (PT), D-dimer, C-reactive protein (CRP), and serum amyloid A (SAA). Conversely, they exhibited lower levels of hemoglobin, platelet count, serum prealbumin, total protein, albumin, uric acid, and lipid parameters (including total cholesterol, HDL, and LDL), as well as reduced electrolytes such as calcium, magnesium, and phosphorus (**Table 1**).

**Table 1:** Comparison of clinical characteristics and admission laboratory indicators between patients with and without Hospital-acquired Infections(HAI).

Categories	HAI group	Non-HAI group	P value
n	213	148	
Sex (n - %)			0.901
Male	135 (63.4)	92 (62.2)	
Female	78 (36.6)	56 (37.8)	
**Age (years)**	**65.00 (55.00-74.00)**	**59.00 (47.00-70.00)**	**0.002**
Underlying diseases (n - %)			
Hypertension	87 (40.8)	67 (45.3)	0.467
Diabetes	31 (14.6)	24 (16.2)	0.777
Malignant tumors/hematological diseases	8 (3.8)	6 (4.1)	1
Kidney injury	35 (16.4)	33 (22.3)	0.206
Hepatitis B	13 (6.1)	6 (4.1)	0.537
**Length of stay (day)**	**46.00 (32.00-65.00)**	**29.00 (17.75-40.50)**	**<0.001**
CBC count			
**White blood cell count (×10^9^/L)**	**11.30 (8.60-13.80)**	**9.75 (7.18-12.75)**	**0.008**
**NLR**	**10.19 (4.78-17.23)**	**6.12 (3.32-13.21)**	**<0.001**
**Hemoglobin (g/L)**	**102.00 (83.00-122.00)**	**115.00 (87.25-128.25)**	**0.012**
Red blood cell distribution width	13.00 (12.40-13.70)	12.85 (12.40-13.60)	0.302
**Platelet count(×10^9^/L)**	**180.00 (126.00-244.00)**	**221.00 (168.00-277.50)**	**<0.001**
Biochemical indicators			
Alanine aminotransferase (U/L)	25.00 (15.00-37.00)	22.00 (14.75-43.50)	0.538
**Aspartate aminotransferase (U/L)**	**35.25 (24.00-52.00)**	**28.00 (19.75-42.25)**	**0.001**
Total Bilirubin (μmol/L)	13.00 (9.20-17.70)	11.30 (8.69-15.88)	0.069
**Prealbumin (mg/dL)**	**18.80 (15.12-23.08)**	**22.20 (17.80-26.20)**	**<0.001**
**Total Protein (g/L)**	**60.50 (54.70-66.50)**	**63.75 (59.00-67.60)**	**<0.001**
**Albumin (g/L)**	**35.30 (31.80-38.70)**	**37.70 (34.40-40.42)**	**<0.001**
Albumin/globulin	1.40 (1.30-1.60)	1.50 (1.30-1.70)	0.159
Creatinine (μmol/L)	61.00 (52.00-76.88)	64.00 (53.00-80.00)	0.392
Urea (mmol/L)	5.56 (4.35-7.30)	5.45 (4.22-7.30)	0.495
**Uric acid (μmol/L)**	**238.00 (183.50-310.62)**	**262.00 (213.00-314.75)**	**0.028**
eGFR(ml/(min·1.73m2))	94.75 (83.75-105.00)	97.00 (82.50-110.00)	0.391
**Serum glucose (mmol/L)**	**7.80 (6.78-9.22)**	**6.91 (5.29-8.72)**	**<0.001**
Serum triglyceride (mmol/L)	1.08 (0.71-1.56)	1.02 (0.63-1.66)	0.493
**Total Cholesterol (mmol/L)**	**3.58 (2.95-4.26)**	**3.94 (3.32-4.52)**	**0.006**
**High-density lipoprotein cholesterol (mmol/L)**	**1.11 (0.92-1.40)**	**1.26 (1.06-1.48)**	**0.001**
**Low density lipoprotein cholesterol (mmol/L)**	**1.79 (1.39-2.37)**	**2.09 (1.54-2.59)**	**0.007**
**Creatine kinase (U/L)**	**314.50 (118.50-796.00)**	**161.00 (66.00-360.50)**	**<0.001**
**Lactate dehydrogenase (U/L)**	**261.00 (211.50-365.00)**	**241.00 (192.00-290.75)**	**0.009**
Serum potassium (mmol/L)	3.91 (3.65-4.22)	3.83 (3.58-4.14)	0.190
Serum sodium (mmol/L)	140.30 (138.10-142.15)	139.50 (138.22-142.10)	0.698
**Serum chlorine (mmol/L)**	**106.30 (103.00-109.47)**	**104.80 (101.95-107.70)**	**0.020**
**Serum calcium (mmol/L)**	**2.10 (1.98-2.21)**	**2.16 (2.07-2.26)**	**<0.001**
**Serum magnesium (mmol/L)**	**0.80 (0.71-0.87)**	**0.82 (0.76-0.89)**	**0.023**
**Serum phosphorus (mmol/L)**	**0.90 (0.73-1.08)**	**0.98 (0.84-1.18)**	**0.011**
Coagulation function			
**Prothrombin time(s)**	**14.40 (13.50-15.39)**	**13.80 (13.00-14.70)**	**<0.001**
**International standardized ratio**	**1.14 (1.04-1.24)**	**1.06 (1.00-1.15)**	**<0.001**
**Fibrinogen (g/L)**	**2.76 (2.15-3.88)**	**3.18 (2.51-4.42)**	**0.001**
**D-dimer (mg/L)**	**8.24 (1.89-18.78)**	**2.06 (0.86-6.48)**	**<0.001**
Blood gas analysis			
pH	7.42 (7.38-7.45)	7.42 (7.38-7.44)	0.175
Oxygenation index (mm/Hg)	358.00 (305.83-419.00)	376.00 (293.00-469.50)	0.475
Plasma lactate (mmol/L)	1.80 (1.20-2.52)	1.50 (1.00-2.35)	0.066
Inflammatory markers			
**C-reactive protein (mg/L)**	**17.20 (3.80-58.90)**	**8.25 (1.95-28.10)**	**0.001**
**Serum amyloid protein A(mg/L)**	**254.15 (102.30-490.45)**	**89.50 (18.30-418.50)**	**0.001**
Erythrocyte Sedimentation Rate (mm/h)	8.00 (2.00-24.00)	8.00 (3.00-17.00)	0.719
Procalcitonin (ng/mL)	0.25 (0.09-0.73)	0.15 (0.08-0.35)	0.090

Bold value indicates that the difference is statistically significant (P < 0.05).

### 3.3 Screening of risk factors for HAI in rehabilitation inpatients

**Figure 3:**
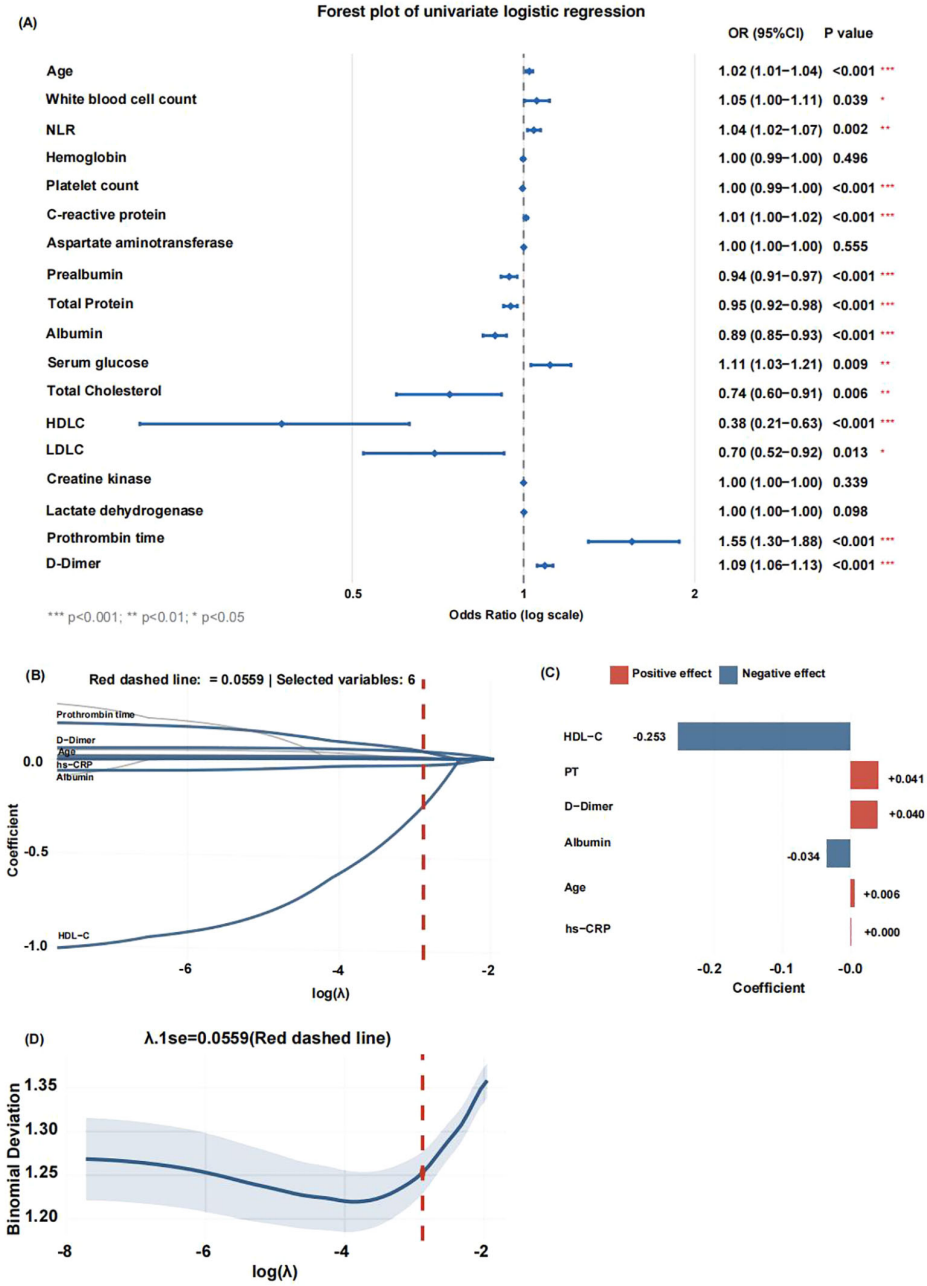
Screening and selection of risk factors for hospital-acquired infections (HAI) in rehabilitation inpatients. **(A)** Univariate logistic regression.**(B)** Variable selection process and coefficient paths in the LASSO regression. The red dashed line indicates the optimal λ value (λ.1se = 0.0559) selected through cross-validation. Six variables (blue) were retained and labeled with their names, while the others (grey) were excluded.**(C)** Coefficient bar plot of the six variables retained in the final model. Red color were identified as risk factors for HAI, whereas blue color served as protective factors. **(D)** Cross-validation error curve illustrating the U-shaped relationship between log(λ) and mean-squared error.

Variables that demonstrated statistically significant differences in intergroup comparisons were first analyzed using univariate logistic regression. The 14 variables screened out by the univariate analysis were subsequently included in a LASSO regression model (**Figure 3A**).

The variable selection process and the coefficient paths are shown in **Figure 3B**. The optimal lambda value (λ.1se = 0.0559) selected via cross-validation is indicated by a red dashed line in the path plot. At this value, which minimizes prediction error while ensuring model parsimony, 6 variables were retained (shown in blue), while the remaining variables were excluded (shown in grey).

The coefficient bar plot (**Figure 3C**) revealed that Age, PT, D-dimer, and CRP were risk factors for HAI, whereas abumin and HDL-C acted as protective factors.

Finally, the cross-validation error curve exhibited a characteristic U-shape (**Figure 3D**). The red dashed line corresponding to λ.1se = 0.0559 is located within one standard deviation of the point with the minimum error, confirming the selection of a robust and parsimonious model.

### 3.4 Development and performance evaluation of a HAI prediction model for rehabilitation inpatients

**Figure 4:**
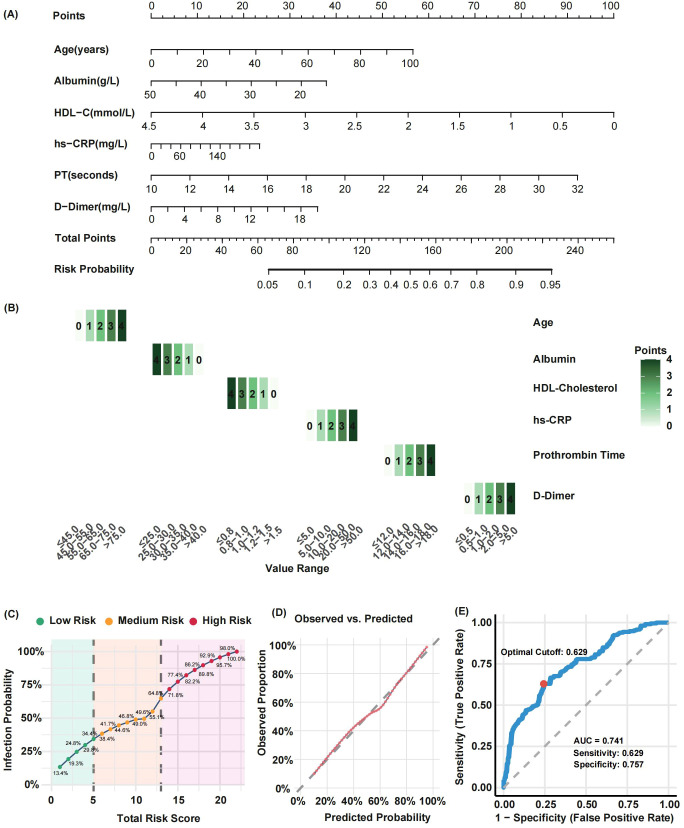
Nomogram model for predicting hospital-acquired infection (HAI) risk in rehabilitation inpatients and its performance evaluation. **(A)** Nomogram constructed based on six variables: Age, prothrombin time (PT), D-dimer, C-reactive protein (CRP), albumin, and high-density lipoprotein cholesterol (HDL-C); **(B)** Heatmap illustrating value intervals and corresponding assigned points for each continuous variable in the final model; **(C)** Risk prediction curve demonstrating the relationship between total score and risk stratification; **(D)** Calibration curve validated by 1000 bootstrap resampling iterations; **(E)** Receiver operating characteristic (ROC) curve evaluating the model’s discriminatory performance.

A nomogram incorporating Age, PT, D-dimer, CRP, Albumin, and HDL-C was developed to predict the risk of HAI (**Figure 4A**). A heatmap clearly illustrates the value intervals and their corresponding assigned points for each continuous variable in the final predictive model (**Figure 4B**). The risk prediction curve demonstrates the relationship between the total score and risk stratification, with defined cut-off points; a higher total score was associated with an increased predicted risk of HAI, showing an upward trend (**Figure 4C**). The calibration curve, validated by 1000 Bootstrap iterations, showed good consistency between the predicted and observed probabilities (**Figure 4D**). The model demonstrated high predictive performance: ROC analysis identified a cut-off value of 0.629, with an AUC of 0.741. The sensitivity and specificity were 62.9% and 75.7%, respectively (**Figure 4E**).

### 3.5 Impact of the COVID-19 pandemic on model stability

A comparison of key predictor variables and infection rates between the pre-pandemic (2018–2019) and pandemic (2020–2025) periods revealed no statistically significant differences in their distributions (**Supplementary Figures 2A–B**).

The predictive model was retrained using pre-pandemic data and validated on pandemic-era data. It demonstrated consistent and robust performance, with the area under the curve (AUC) remaining stable at approximately 0.708 (**Supplementary Figure 2C**).

### 3.6 External validation of the HAI prediction model for rehabilitation inpatients

**Figure 5:**
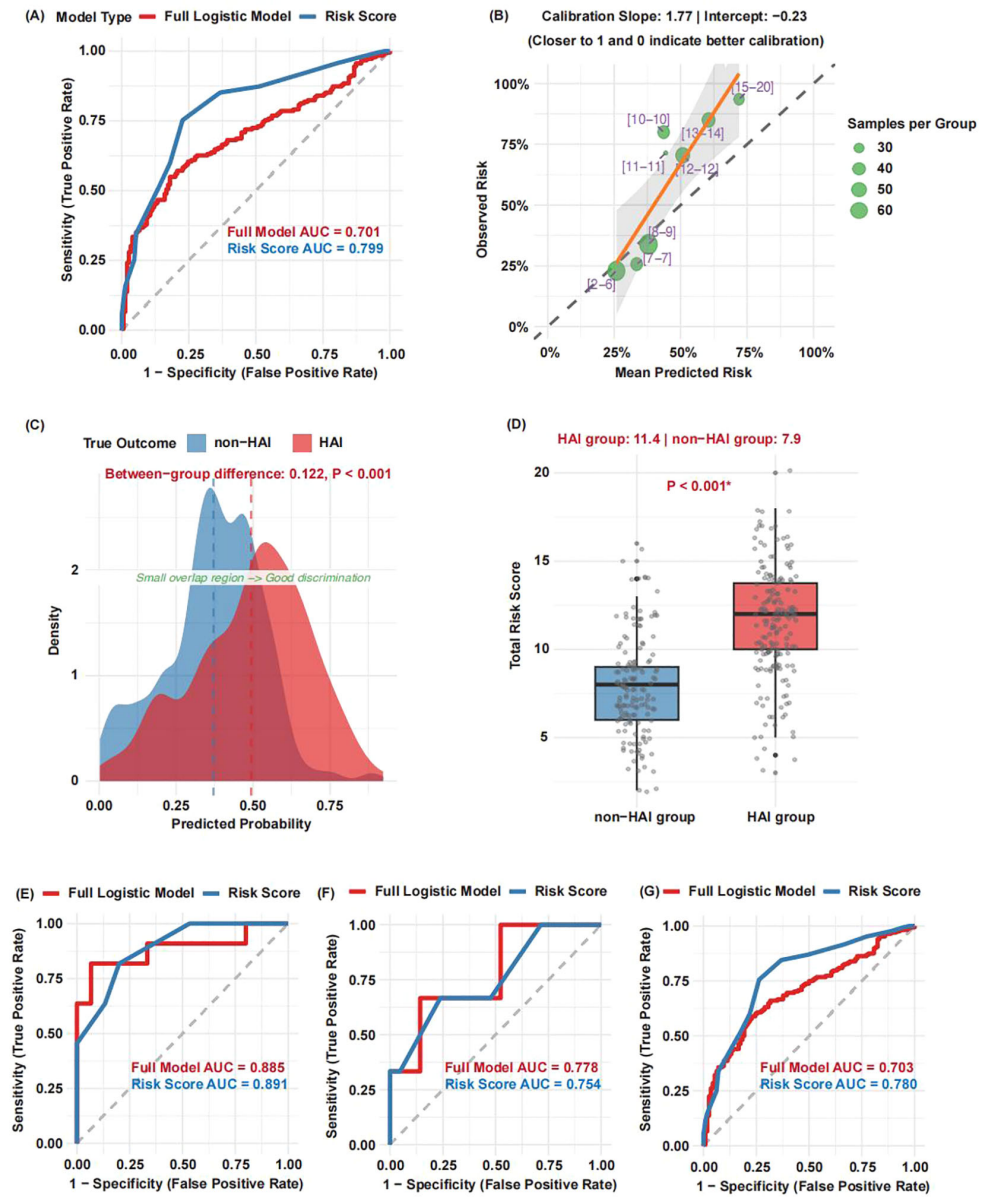
External validation of the HAI prediction model in rehabilitation inpatients. **(A)** Receiver operating characteristic (ROC) curves for the full logistic regression model and the simplified risk score; **(B)** Calibration curve across eight risk groups, an ideal slope is 1.0 and an ideal intercept is 0.0; **(C)** Density plots of predicted probabilities for HAI and non-HAI patients; **(D)** Comparison of risk scores between HAI and non-HAI patients. **(E–G)** Model performance on three independent external validation sub-cohorts.

The external validation cohort comprised 332 patients from three independent hospitals, with an observed infection rate of 54.8%. ROC analysis demonstrated strong discriminatory performance for both the full logistic regression model (AUC = 0.701) and the simplified risk score (AUC = 0.799) (**Figure 5A**). Calibration across eight risk groups yielded a slope of 1.77 (ideal = 1.0) and an intercept of –0.23 (ideal = 0.0), indicating acceptable overall calibration with a tendency toward slight overestimation in higher-risk ranges (**Figure 5B**).

Density plots revealed clear separation in predicted probabilities between HAI and non-HAI patients (mean difference = 0.122, P < 0.001) (**Figure 5C**). Consistently, HAI patients exhibited significantly higher risk scores (mean = 11.4) than non-HAI patients (mean = 7.9; P < 0.001) (**Figure 5D**).

The evaluations separately on the three independent validation sub-cohorts, the model showed robust performance: the full logistic regression model achieved AUCs of 0.885, 0.778, and 0.703, while the simplified risk score attained AUCs of 0.891, 0.754, and 0.780, respectively (**Figures 5E–G**).

## 4 Discussion

This study systematically evaluated the dynamic characteristics and drug resistance of microbial culture in 635 inpatients in the department of rehabilitation, and revealed the epidemiological model, risk factors and the effectiveness of the prediction model of hospital−acquired infection.

The study revealed a markedly higher microbial culture positivity rate of 61.2% in rehabilitation patients, with respiratory samples demonstrating particularly elevated rates (sputum 80.0%, BALF 70.0%). These findings substantially exceed reported rates in critically ill patients (31.4%) (Wu et al., 2021), community-acquired pneumonia cases (52.9%) (Uzer et al., 2024), and severe infection cohorts (BALF 25.0%) (Hou et al., 2025). The observed disparity likely stems from rehabilitation patients’ distinctive demographic profile - predominantly elderly individuals with chronic conditions who exhibit immunocompromised states. The dominance of respiratory infections, corroborated by prior research (Uzer et al., 2024), underscores the clinical imperative for prioritizing respiratory sample monitoring to enhance early detection and management of infectious diseases in this vulnerable population.

Time-series analysis revealed site-specific pathogen dynamics in hospitalized patients. Respiratory infections were initially dominated by *Klebsiella pneumoniae*, aligning with community-acquired pneumonia patterns (Koo et al., 2021), whereas *Pseudomonas aeruginosa* prevalence increased with prolonged hospitalization, correlating with ventilator use and broad-spectrum antibiotic exposure (Guillon et al., 2021). Bloodstream infections followed a sequential pattern: *Staphylococcus* predominated in the first week (catheter-related), succeeded by *Serratia marcescens* in week 2 to week 3 (central venous catheter-associated) (Cappelletti et al., 2024), and later by fungi and *Stenotrophomonas maltophilia* in week 4 to week 5(driven by antibiotic-induced dysbiosis) (Erdiren and Kula Atik, 2025). Concurrently, cerebrospinal fluid co-infections (*Staphylococcus* + *Enterobacter aerogenes*) and urinary tract pathogen shifts (from *Enterobacteriaceae* to *Enterococcus faecium/Escherichia coli*) were linked to surgical procedures and catheterization (Zelnik Yovel et al., 2021). These findings support stage-specific interventions: early empirical coverage for *Klebsiella pneumoniae* in respiratory cases, intensified *Pseudomonas aeruginosa* surveillance in later stages, and weekly-adjusted bloodstream infection protocols with fungal coverage after week 4, alongside optimized catheter management to reduce invasive procedure risks.

This study demonstrated a significant positive correlation between the duration of hospitalization and the risk of nosocomial infections. Specifically, the urine culture positivity rate rose to 75% after four weeks of hospitalization, while the blood culture positivity rate continued to increase beyond six months, indicating that prolonged hospital stays are a major risk factor for both urinary tract infections and bacteremia. Previous studies have suggested that extended hospitalization elevates the risk of enterococcal-associated complicated urinary tract infections by contributing to malnutrition (e.g., reduced serum albumin) and immunosuppression (Niseteo et al., 2020), compounded by the cumulative exposure to invasive procedures such as indwelling catheterization. This effect appears synergistic with hypoproteinemia (Zhu et al., 2025). Therefore, we recommend that rehabilitation departments adopt a phased infection control strategy, focusing on respiratory infections in the early stages and shifting attention to urinary tract and bloodstream infections in later stages.

In terms of antimicrobial resistance, our cohort revealed high resistance rates among Enterobacteriaceae to β-lactam/β-lactamase inhibitor combinations (e.g., ampicillin and ticarcillin/clavulanic acid). Resistance to penicillin was widespread in *Staphylococcus* spp., with coagulase-negative staphylococci (CoNS) exhibiting broad resistance to quinolones, macrolides, and clindamycin. *Pseudomonas aeruginosa* showed elevated resistance to carbapenems (imipenem) and β-lactam/β-lactamase inhibitors. In clinical practice, especially in the absence of clear etiological evidence, empirical use of broad-spectrum antibiotics remains common, directly selecting for and facilitating the survival and spread of resistant strains (Savic et al., 2025). This context highlights the need to develop predictive models that integrate clinical and laboratory data to enable early identification of patients at risk for hospital−acquired infection. Such a prevention-oriented approach would help reduce empirical use of broad-spectrum antibiotics and slow the progression of antimicrobial resistance.

Our analyses identified age, prothrombin time, D-dimer, and C-reactive protein as significant risk factors for hospital−acquired infection in rehabilitation inpatients, whereas albumin and high-density lipoprotein cholesterol emerged as protective factors. These biomarkers collectively reflect critical underlying pathophysiological processes: immunosenescence and innate immune dysfunction (age) (He et al., 2021; Alhazmi et al., 2025); coagulation cascade activation and endothelial injury (prolonged PT and elevated D-dimer) (Mihaila and Dragos Mihaila, 2020; Naqvi et al., 2022; Schafer et al., 2022; Zhang et al., 2023); and impaired antimicrobial defense and systemic inflammation (hypoalbuminemia and reduced HDL-C) (Rao et al., 2021; Schafer et al., 2022; Chen et al., 2024). The identified risk profile is supported by existing evidence linking these parameters to infection susceptibility and severity in conditions such as severe pneumonia, COVID-19, and sepsis. Consequently, integrating assessment of these readily available markers could enhance risk stratification and inform targeted preventive strategies in the vulnerable rehabilitation inpatient population.

We developed a nomogram incorporating six clinically accessible biomarkers, which demonstrated consistent performance throughout the COVID-19 pandemic. This suggests that the core biological mechanisms driving infection risk remain stable despite temporal variations in pathogens or clinical protocols. External validation across three independent hospitals confirmed the model’s robustness, with a simplified risk score achieving an area under the curve (AUC) of 0.799. Although slight overestimation occurred in higher-risk ranges, the overall calibration was acceptable. Moreover, the combination of a nomogram and risk score enhances clinical utility by facilitating rapid risk stratification and guiding targeted interventions—such as intensified surveillance, early mobilization, and nutritional support—for high-risk patients. In contrast to complex multi-omics models (Gong et al., 2025; Rao et al., 2025), our tool is readily deployable even in resource-limited settings.

This study presents two key innovations in rehabilitation medicine: (1) integrated six conventional and easily available indicators to construct a special model for the specific scenario of rehabilitation department; (2) developed a user-friendly, openly accessible online risk calculator to facilitate clinical application, available at: https://wjing-enzemed.shinyapps.io/hospital-infection-risk-en/.

## 5 Limitation

This study underscores the utility of routine laboratory parameters for infection risk prediction but is constrained by its retrospective design—which may introduce selection and measurement biases—along with incomplete data and irregular sampling intervals affecting infection timeline accuracy. The model does not incorporate key clinical confounders, such as antimicrobial history, invasive procedure details, functional scores, or device indwelling duration, nor was it prospectively validated. Future research should prioritize standardized prospective validation and the integration of both laboratory and clinical predictors to enhance model comprehensiveness and applicability in real-world settings.

## 6 Conclusion

In summary, this rehabilitation-specific prediction model provides an objective tool for early hospital−acquired infection risk identification, facilitating targeted prevention to shorten hospitalization, reduce costs, and improve outcomes. Further multicenter validation is warranted before broad clinical adoption.

## Data Availability

The raw data supporting the conclusions of this article will be made available by the authors, without undue reservation.

## Supplementary material

Supplementary Figure 1 Pathogen distribution and antimicrobial resistance profiles in rehabilitation inpatients. **(A)** Antimicrobial resistance patterns of the most frequently isolated bacterial pathogens; **(B)** Resistance rates of MDRO carriers and non-MDRO carriers; **(C)** Overall resistance rates were observed across specimen types; **(D)** Prevlences of MDRO types.

Supplementary Figure 2 Impact of the COVID-19 pandemic on the stability of the HAI prediction model. **(A)** Comparison of key predictor variables between the pre-pandemic (2018–2019) and pandemic (2020–2025) periods. **(B)** Comparison of infection rates between the pre-pandemic (2018–2019) and pandemic (2020–2025) periods. **(C)** Performance of the predictive model retrained on pre-pandemic data and validated on pandemic-era data.
