# Supplementary figures and images for: From microbial dynamics to risk prediction: a nomogram-based model for hospital-acquired infections in rehabilitation settings
Source: Front Cell Infect Microbiol. 2026 Feb 17;16:1723835. doi: 10.3389/fcimb.2026.1723835 (PMC12953564; doi:10.3389/fcimb.2026.1723835)

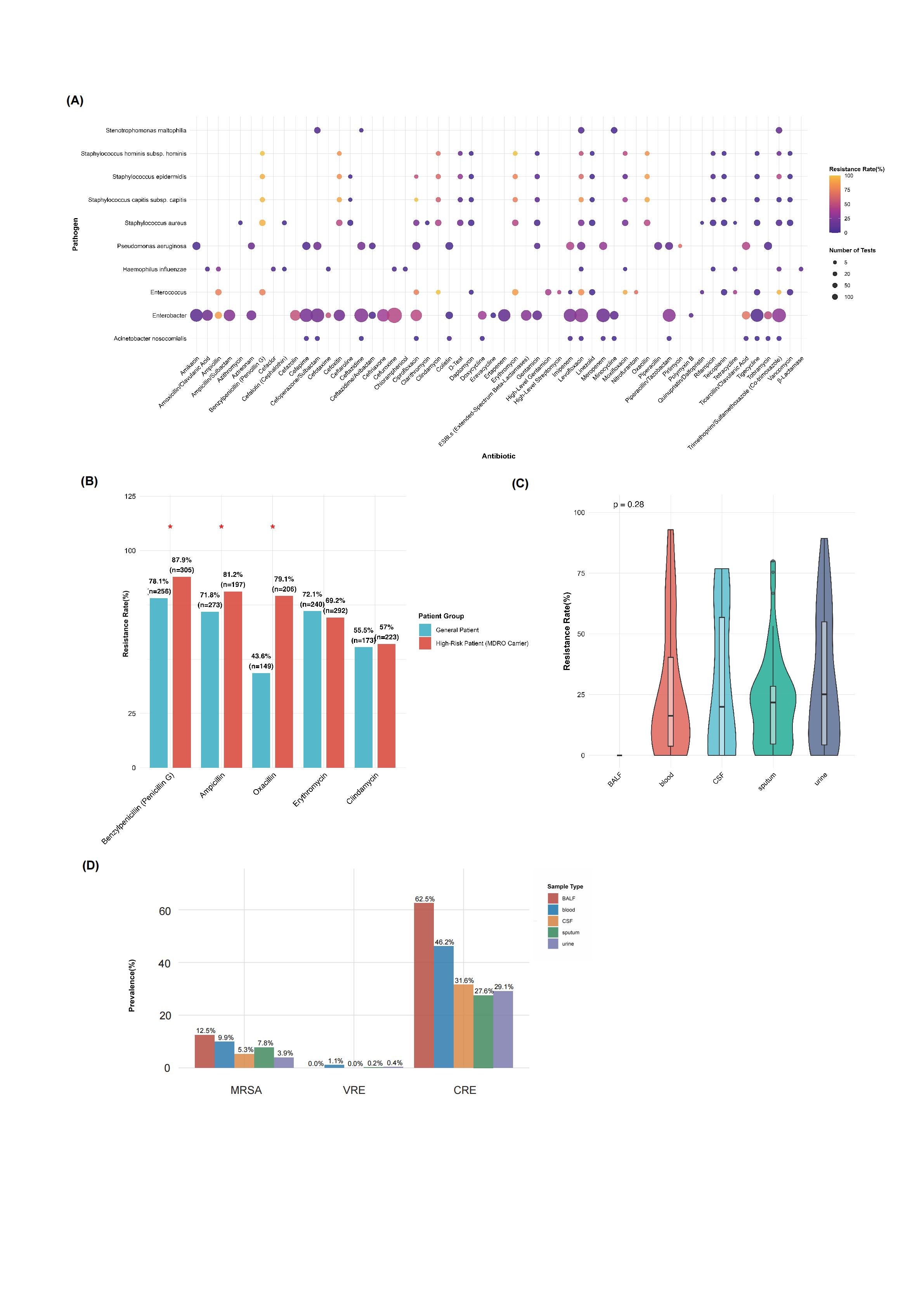

Supplement: Supplementary Figure 1 — Pathogen distribution and antimicrobial resistance profiles in rehabilitation inpatients. (A) Antimicrobial resistance patterns of the most frequently isolated bacterial pathogens; (B) Resistance rates of MDRO carriers and non-MDRO carriers; (C) Overall resistance rates were observed across specimen types; (D) Prevlences of MDRO types. [file Image1.jpeg]

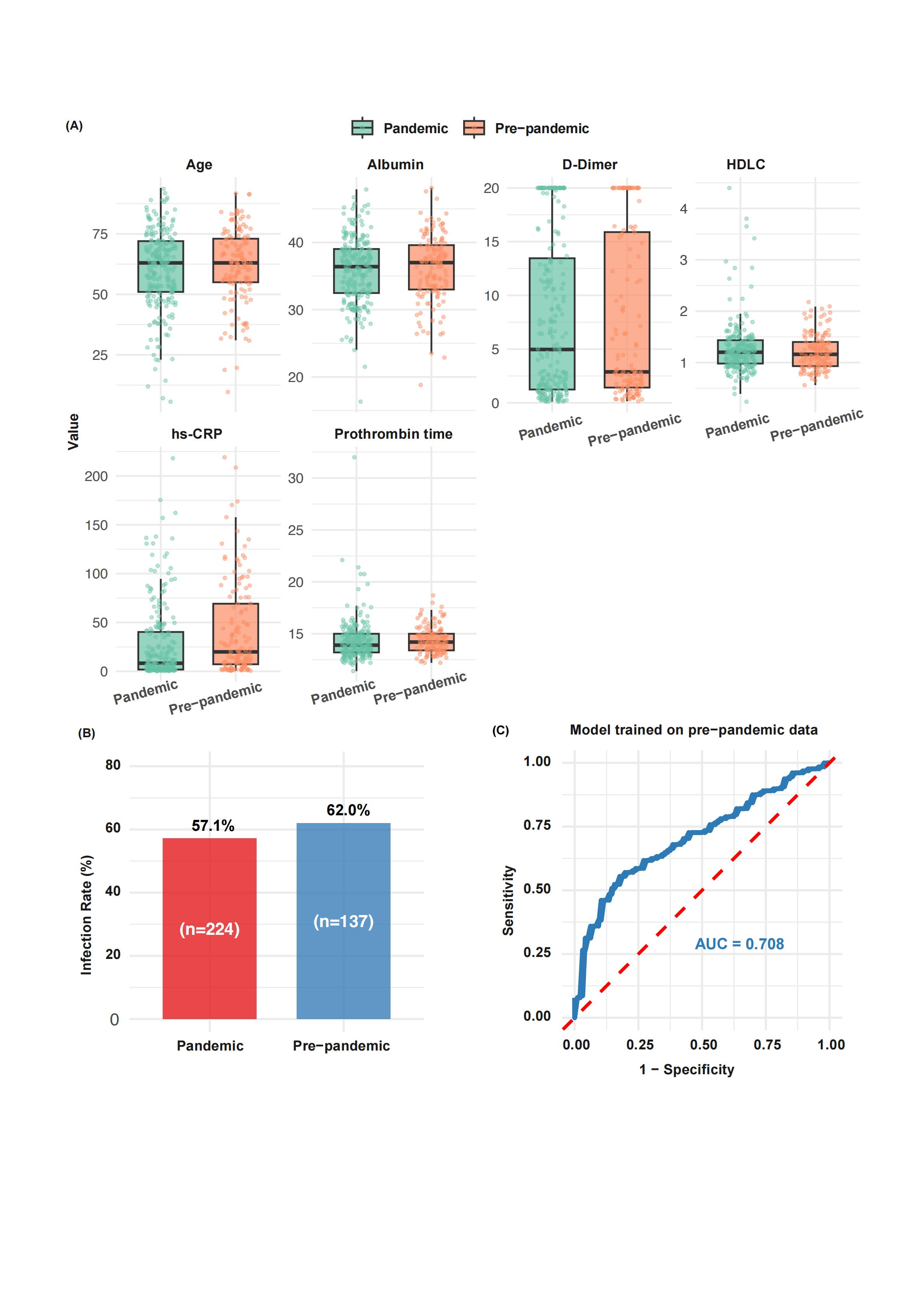

Supplement: Supplementary Figure 2 — Impact of the COVID-19 pandemic on the stability of the HAI prediction model. (A) Comparison of key predictor variables between the pre-pandemic (2018–2019) and pandemic (2020–2025) periods. (B) Comparison of infection rates between the pre-pandemic (2018–2019) and pandemic (2020–2025) periods. (C) Performance of the predictive model retrained on pre-pandemic data and validated on pandemic-era data. [file Image2.jpeg]
